# Hospitalization, case fatality, comorbidities, and isolated pathogens of adult inpatients with pneumonia from 2013 to 2022: a real-world study in Guangzhou, China

**DOI:** 10.1186/s12879-023-08929-y

**Published:** 2024-01-02

**Authors:** Yun Li, Zhufeng Wang, Lunfang Tan, Lina Liang, Shuyi Liu, Jinhai Huang, Junfeng Lin, Kang Peng, Zihui Wang, Qiasheng Li, Wenhua Jian, Baosong Xie, Yi Gao, Jinping Zheng

**Affiliations:** 1 National Center for Respiratory Medicine, National Clinical Research Center for Respiratory Disease, State Key Laboratory of Respiratory Disease, Guangzhou Institute of Respiratory Health, The First Affiliated Hospital of Guangzhou Medical University, Guangzhou, China; 2 Department of Pulmonary and Critical Care Medicine, Fujian Provincial Hospital, Fujian Medical University, Fuzhou, China

**Keywords:** Pneumonia, Hospitalization, Mortality, Comorbidity, Natural language processing

## Abstract

**Background:**

In the context of increasing population aging, ongoing drug-resistant pathogens and the COVID-19 epidemic, the changes in the epidemiological and clinical characteristics of patients with pneumonia remain unclear. This study aimed to assess the trends in hospitalization, case fatality, comorbidities, and isolated pathogens of pneumonia-related adult inpatients in Guangzhou during the last decade.

**Methods:**

We retrospectively enrolled hospitalized adults who had doctor-diagnosed pneumonia in the First Affiliated Hospital of Guangzhou Medical University from January 1, 2013 to December 31, 2022. A natural language processing system was applied to automatically extract the clinical data from electronic health records. We evaluated the proportion of pneumonia-related hospitalizations in total hospitalizations, pneumonia-related in-hospital case fatality, comorbidities, and species of isolated pathogens during the last decade. Binary logistic regression analysis was used to assess predictors for patients with prolonged length of stay (LOS).

**Results:**

A total of 38,870 cases were finally included in this study, with 70% males, median age of 64 (53, 73) years and median LOS of 7.9 (5.1, 12.8) days. Although the number of pneumonia-related hospitalizations showed an upward trend, the proportion of pneumonia-related hospitalizations decreased from 199.6 per 1000 inpatients in 2013 to 123.4 per 1000 in 2021, and the case fatality decreased from 50.2 per 1000 in 2013 to 23.9 per 1000 in 2022 (all *P* < 0.05). The most common comorbidities were chronic obstructive pulmonary disease, lung malignancy, cardiovascular diseases and diabetes. The most common pathogens were *Pseudomonas aeruginosa*, *Candida albicans*, *Acinetobacter baumannii*, *Stenotrophomonas maltophilia*, *Klebsiella pneumoniae*, and *Staphylococcus aureus.* Glucocorticoid use during hospitalization (Odd Ratio [OR] = 1.86, 95% Confidence Interval (CI): 1.14–3.06), immunosuppressant use during hospitalization (OR = 1.99, 1.14–3.46), ICU admission (OR = 16.23, 95%CI: 11.25–23.83), receiving mechanical ventilation (OR = 3.58, 95%CI: 2.60–4.97), presence of other underlying diseases (OR = 1.54, 95%CI: 1.15–2.06), and elevated procalcitonin (OR = 1.61, 95%CI: 1.19–2.19) were identified as independent predictors for prolonged LOS.

**Conclusion:**

The proportion of pneumonia-related hospitalizations and the in-hospital case fatality showed downward trends during the last decade. Pneumonia inpatients were often complicated by chronic underlying diseases and isolated with gram-negative bacteria. ICU admission was a significant predictor for prolonged LOS in pneumonia inpatients.

**Supplementary Information:**

The online version contains supplementary material available at 10.1186/s12879-023-08929-y.

## Introduction

Pneumonia is a common respiratory disease with high morbidity and mortality in all age groups worldwide [1]. The latest Global Burden of Disease (GBD) study showed that lower respiratory tract infections (LRTIs) including pneumonia and bronchitis affected 489 million people globally [2]. The mortality of LRTIs has declined significantly during the past 30 years due to advances in anti-infective drugs, but it remains an important health issue for individuals, especially for the children and the elderly [2]. In the context of increasing population aging and ongoing drug-resistant pathogens, some changes may have occurred in the inpatients with pneumonia. Furthermore, in recent years, the pandemic of coronavirus disease 2019 (COVID-19) has had a great impact on people’s health status, medical treatment and living habits, but its role in the epidemiological trends of pneumonia is not known definitively. However, there is still a lack of relevant research.

Real-world data, referring to any clinical data other than traditional clinical trials [3], have some advantages such as strong external authenticity, wide sources and large sample size, and have been applied in clinical decision-making, hospital management and medical insurance. The electronic health record (EHR) system, widely used in healthcare institutions, is an important carrier of real-world data [4]. However, since the EHR data are vast, unstructured, multidimensional and scattered, and have random errors and systematic bias [5], utilization of EHR remains a great challenge. Notably, processing EHR data by artificial intelligence (AI) and natural language processing (NLP) have been gaining increasing attention. For example, previous studies showed that the AI models constructed by NLP had similar diagnostic accuracy for common pediatric diseases compared with an experienced pediatrician [6] and have been applied to detect postoperative complications in hospitalized patients [7], screen for various types of drug abuse [8], identify the symptoms in maintenance hemodialysis patients [9] and predict serious adverse events during maternal delivery [10]. Similarly, our center has developed a big data platform with a NLP system to automatically extract the targeted EHR data of patients. As reported previously, our colleagues employed this platform to analyze the trends of pulmonary fungal infections in Guangzhou from 2013 to 2019 [11] and identify pulmonary aspergillosis cases with defects in diagnosis and treatment [12].

Therefore, we want to continue to leverage the advantages of AI and NLP in dealing with vast amounts of real-world data about inpatients with pneumonia. The primary objective of this study was to assess the trends in pneumonia-related hospitalizations, case fatality, comorbidities, and isolated pathogens in Guangzhou during the last decade. The secondary objective was to assess the potential predictors for prolonged length of hospital stay.

## Materials and methods

This was a retrospective study, and data were derived from the big data platform of National Center for Respiratory Medicine, the First Affiliated Hospital of Guangzhou Medical University. A NLP system was applied to automatically extract the clinical data from EHRs via the big data platform.

### Study population and the extracted information

We retrospectively enrolled inpatients who were discharged in the First Affiliated Hospital of Guangzhou Medical University from January 1, 2013 to December 31, 2022. The main inclusion criteria were patients who aged≥18 years and had doctor-diagnosed pneumonia or pulmonary infection. Doctor-diagnosed pneumonia or pulmonary infection was based on the widely used criteria: (1) presence of a new pulmonary infiltrate on chest X-ray or computed tomography scan at the time of hospitalization; (2) at least one of the following (a) new cough or increased cough or sputum production, (b) fever, (c) leucocyte count > 10,000 cells/mm^3^ or < 4000 cells/mm^3^. The exclusion criteria encompassed patients who were medically diagnosed with non-infectious pneumonia, such as radiation pneumonia, and interstitial pneumonia, as well as those with incomplete essential parameters. The information to be extracted included: personal information (gender, age, height, weight, and smoking history); respiratory comorbidities or other underlying diseases, including chronic obstructive pulmonary disease (COPD), asthma, bronchiectasis, pleural effusion, tuberculosis, lung malignancy, interstitial lung disease, pulmonary embolism, hypertension, diabetes, coronary heart disease, stroke, connective tissue disease, chronic kidney disease, and hematological malignancy; etiological data (bacteria, fungi, and viruses); inflammatory indicators such as leukocytes, hypersensitive C-reactive protein, sedimentation, erythrocyte sedimentation rate, and procalcitonin; use of glucocorticoid or immunosuppressive drugs during hospitalization; discharge outcomes; length of stay (LOS); and hospitalization cost.

### Data production and extraction process

**Fig. 1:**
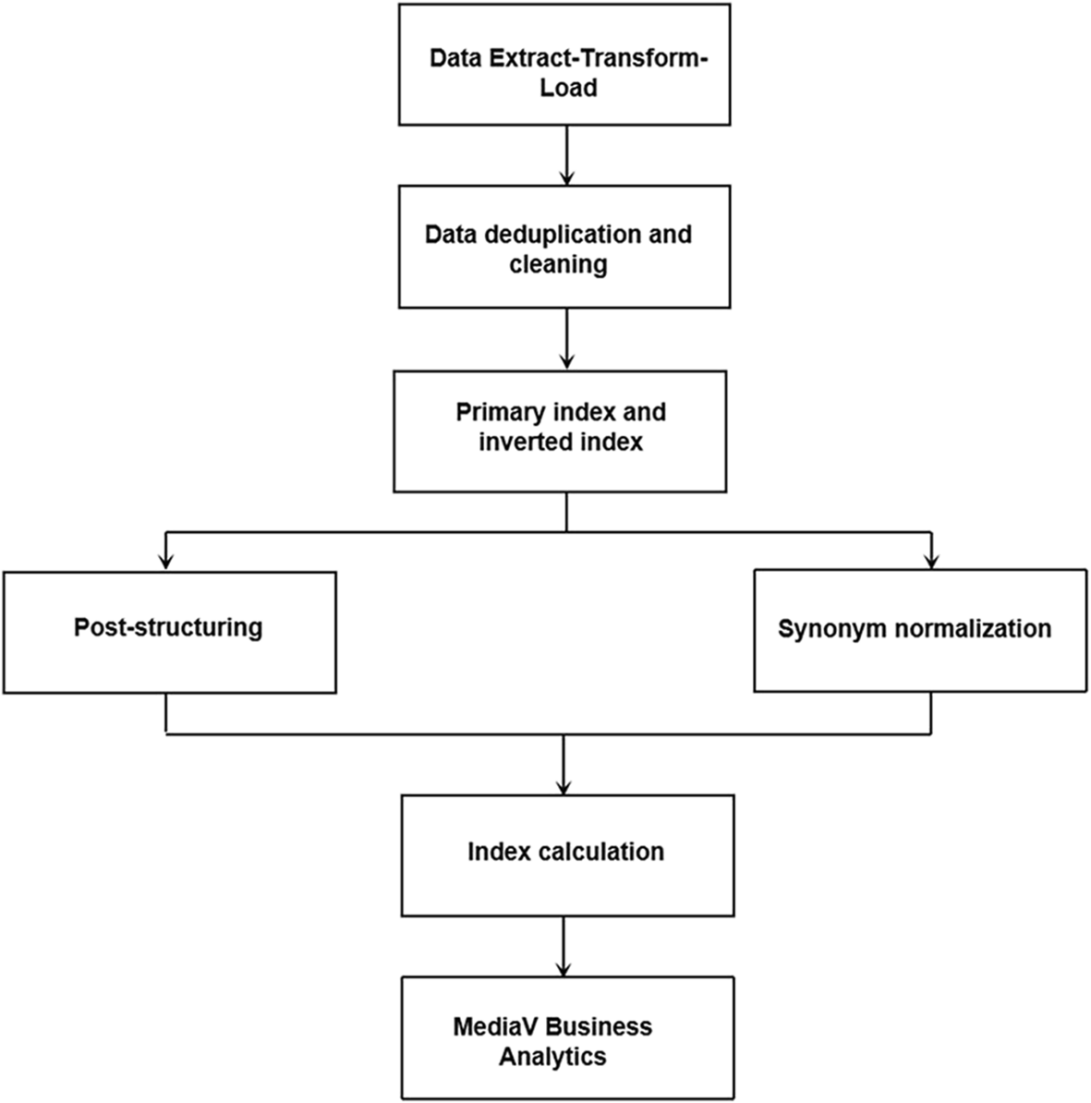
The process of data production and extraction

All data collection was authorized by the relevant departments of the hospital. As shown in Fig. 1, in the Extract-Transform-Load (ETL) step, we use Kettle (an open-source ETL tool) to write scripts such as extraction, conversion, and loading and then collect relevant data from various business systems of hospitals to the big data platform. If there are duplicate records in the collected data, deduplication will be carried out. Data that do not meet the requirements of the interface will be discarded. If the data is partially missing, it may be supplemented through some certain associations. For example, if the age is missing during information collection, it can be acquired by transforming the birthday. Primary index, in short, is the basic information retrieval directory of a patient. After calculation of the patient primary index, a primary index field is added to back-brush the data and correlate all the data on the same person. Post-structuring is the automatic identification and conversion of unstructured medical texts such as inpatient medical records, inspection reports, and prescriptions into structured data. Synonym normalization refers to the classification of short text data, for instance, one medical term may have multiple names and needs synonym normalization. Index calculation is mainly to unify the standardized results which have been processed with post-structuring and synonym normalization. MediaV Business Analytics, an important step in the construction of database, is used to find the characteristics of the population by the aggregate analysis. Specific methods have been published in a previous study [11].

In the process of data collection, we strictly follow data security standards. In order to ensure the security of data throughout the process, the construction of the platform complies with the national “three-level information security protection” certification and “ISO27001” certification standards. We conducted in-depth data verification of the entire process of data production, and provided a data verification report to ensure the accuracy, authenticity and reliability of the extracted data.

### Data analysis

After data cleaning and sorting, we evaluated the trends in the gender proportion, age stratification, number of pneumonia-related hospitalizations, proportion of pneumonia-related hospitalizations in total hospitalizations, number of pneumonia-related deaths, in-hospital case fatality, and proportions of pneumonia patients with pulmonary diseases or other underlying diseases during the last 10 years. We also analyzed the trends in the percentage of different isolated pathogens and then performed the pair-to-pair interaction. The proportion of pneumonia-related hospitalizations in total hospitalizations = (the number of hospitalized patients with pneumonia per year)/(the total number of hospitalized patients per year)*1000. The pneumonia-related in-hospital case fatality = (the number of pneumonia-related deaths per year)/(the number of pneumonia-related hospitalizations per year)*1000. The percentage of one isolated pathogen = (the number of one isolated pathogen per year)/(total number of all isolated pathogens per year)*100%. The positive rate of virus detection = (the number of positive cases of one particular virus)/(total number of the cases that being tested for this virus).

All patients with pneumonia were divided into two groups according to the LOS: prolonged group (LOS ≥ median + 2SD days) and non-prolonged group (LOS < median + 2SD days). Since the number of cases in non-prolonged group was much larger than that in prolonged group and may cause bias, a random sample from non-prolonged group was extracted at a 2:1 ratio of non-prolonged group to prolonged group. Then the total set was divided into a training set and a validation set at a ratio of 7:3. Binary logistic regression analysis was used to examine potential predictors with prolonged LOS in patients. The variables considered in this analysis included age, gender (female or male), smoking history (never smoking, ever smoking, or current smoking), glucocorticoid use during hospitalization (yes or no), immunosuppressant during hospitalization (yes or no), ICU admission (yes or no), receiving mechanical ventilation (yes or no), presence of pulmonary fungal infection (yes or no), presence of respiratory comorbidities (yes or no), presence of other underlying diseases (yes or no), serum albumin (normal or decreased), and procalcitonin (normal or elevated).

### Statistical analysis

Analysis was performed using R (version 4.0.2). Continuous data were expressed as median (Q_25_, Q_75_), and categorical data were expressed as frequency (percentage). General linear regression method was used to analyze the trends of each index over the years. All the above tests were bilateral, the test level α = 0.05, P<0.05 was considered statistically significant.

## Results

### An overview of clinical data in patients with pneumonia

A total of 65,443 cases were initially extracted from the big data platform. After excluding the cases with a discharge diagnosis unrelated to pneumonia or lung infection (*n* = 19,837), and those who were diagnosed with non-infectious pneumonia (*n* = 6836), 38,870 cases were included in the final analysis. These cases had 70% males, with a median age of 64 (53, 73) years, a median LOS of 7.9 (5.1, 12.8) days, a median hospitalization cost of 19,107.2 (12,127.0, 34,275.0) yuan, and 2359 ICU admissions. The majority (80%) of these patients were admitted to the department of respiratory medicine, and 35% were ever-smokers or current smokers (Table 1).

**Table 1:** An overview of clinical data in inpatients with pneumonia (*n* = 38,870)

Variable	Value, n (%) or median (Q_25_, Q_75_)
**Department**	
Respiratory medicine	31,219 (80)
Other internal medicine	5731 (15)
Surgery	1920 (5)
**Sex**	
Female	11,846 (30)
Male	27,024 (70)
**Age (year)**	64 (53, 73)
**BMI (kg/m**^**2**^**)**	21.6 (19.0, 24.2)
**Smoking history**	
Never smoker	25,254 (65)
Ever smoker	6797 (17)
Current smoker	6819 (18)
**Glucocorticoid use during hospitalization**	
No	22,442 (58)
Yes	16,428 (42)
**Immunosuppressant use during hospitalization**	
No	19,799 (51)
Yes	19,071 (49)
**Cost (yuan)**	19,107.2 (12,127.0, 34,275.0)
**Length of stay (day)**	7.9 (5.1, 12.8)
**ICU admission**	
No	36,511 (94)
Yes	2359 (6)
**Receiving mechanical ventilation during hospital**	
No	25,267 (65)
Yes	13,603 (35)
**Discharge outcome**	
Unknown	4786 (12)
Improved	31,833 (82)
Worsen	950 (2)
Dead	1301 (3)
**Pulmonary fungal infection**	
No	33,526 (86)
Yes	5344 (14)
**Viral pneumonia**	
No	38,412 (99)
Yes	458 (1)
**Albumin (g/L)**	35.0 (31.3, 38.2)
White blood cell (*10^**9**^**/L)**	8.1 (6.1, 11.0)
**Erythrocyte sedimentation rate (mm/h)**	46 (20, 85)
**Hypersensitive C-reactive protein (mg/L)**	20.5 (3.32, 71.2)
**Procalcitonin**	
Normal	10,828 (48)
Elevated	11,758 (52)
Missing	16,284

### Trends in gender and age composition

From the year 2013 to 2022, the proportion of males was higher than that of females each year (Supplementary Fig. 1). The age composition did not show significant changes in general and the proportion of patients aged ≥58 years old accounted for about 62% ~ 70% each year (Table 2). A slight increase was observed in the proportion of patients aged 38–58 years old during the last decade (*P* < 0.01), while a slight decrease was observed in the proportion of patients aged ≥78 years old (*P* < 0.01) (Supplementary Fig. 2).

**Table 2:** Trends in gender and age composition of inpatients with pneumonia from 2013 to 2022, n (%)

Variable	Total (n = 38,870)	2013 (*n* = 3086)	2014 (*n* = 3223)	2015 (*n* = 3047)	2016 (*n* = 3555)	2017 (*n* = 3426)	2018 (*n* = 4056)	2019 (*n* = 4872)	2020 (*n* = 3820)	2021 (*n* = 4183)	2022 (*n* = 5602)
**Sex**											
Female	11,846 (30)	991 (32)	965 (30)	872 (29)	1080 (30)	956 (28)	1220 (30)	1450 (30)	1153 (30)	1289 (31)	1870 (33)
Male	27,024 (70)	2095 (68)	2258 (70)	2175 (71)	2475 (70)	2470 (72)	2836 (70)	3422 (70)	2667 (70)	2894 (69)	3732 (67)
**Age (year)**											
Median (Q_25_, Q_75_)	64 (53, 73)	65 (54, 76)	66 (55, 76)	65 (54, 75)	64 (53, 75)	64 (54, 75)	64 (54, 74)	64.5 (53, 73)	63 (51, 71)	63 (52, 71)	62 (52, 70)
**Age group**											
> = 18 to < 38	3428 (9)	275 (9)	242 (8)	234 (8)	300 (8)	284 (8)	349 (9)	437 (9)	386 (10)	368 (9)	553 (10)
> = 38 to < 58	9685 (25)	720 (23)	724 (22)	702 (23)	808 (23)	766 (22)	963 (24)	1204 (25)	1067 (28)	1155 (28)	1576 (28)
> = 58 to < 78	19,561 (50)	1498 (49)	1612 (50)	1490 (49)	1787 (50)	1739 (51)	2032 (50)	2463 (51)	1877 (49)	2162 (52)	2901 (52)
> = 78	6196 (16)	593 (19)	645 (20)	621 (20)	660 (19)	637 (19)	712 (18)	768 (16)	490 (13)	498 (12)	572 (10)

### Trends in pneumonia-related hospitalizations and in-hospital case fatality

**Fig. 2:**
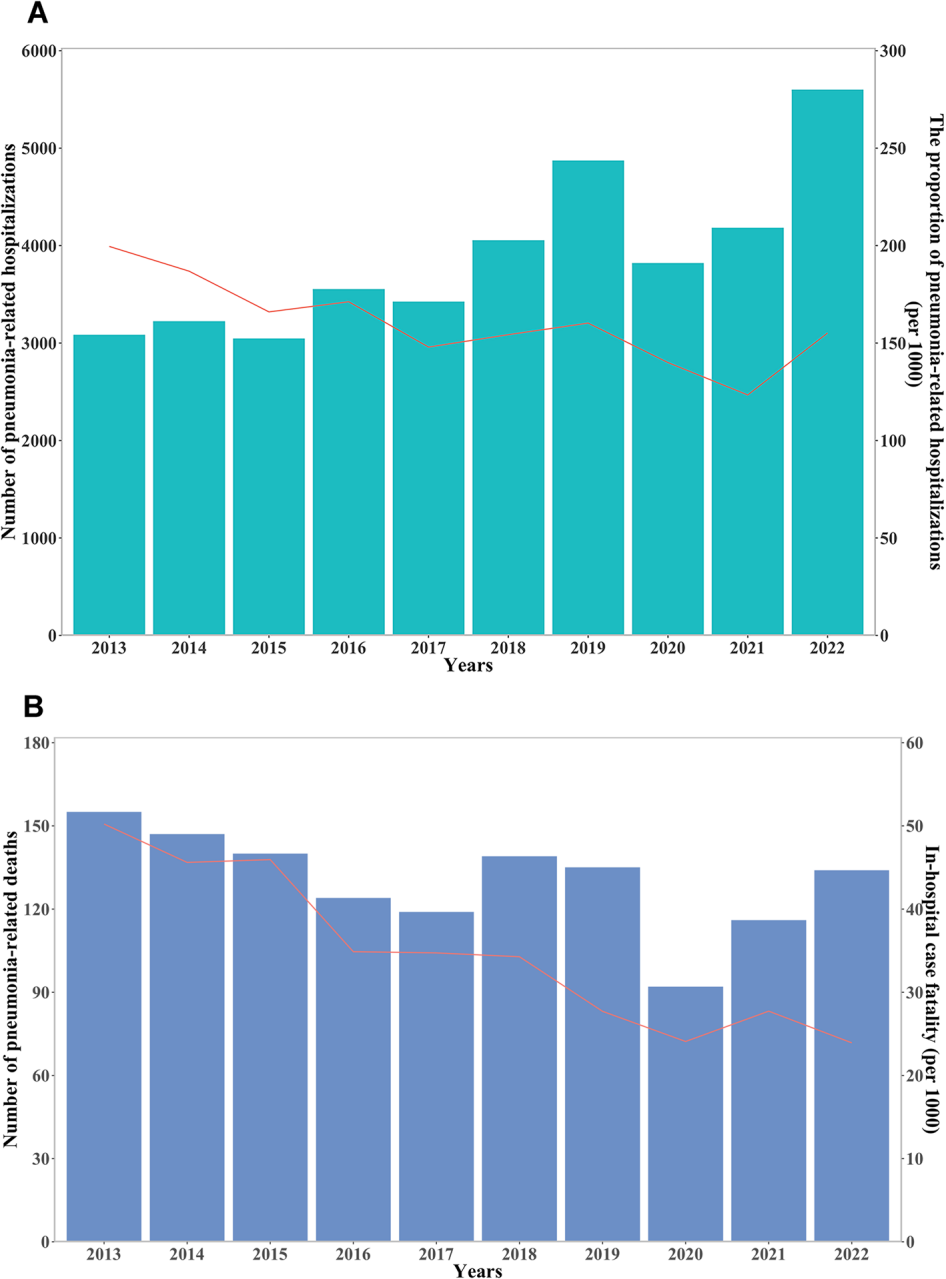
The trends in the number of pneumonia-related hospitalizations and the proportion of pneumonia-related hospitalizations in total hospitalizations **A**; the number of pneumonia-related deaths and in-hospital case fatality **B**

An upward trend was observed in the absolute number of pneumonia-related hospitalizations, whereas a downward trend was shown in the proportion of pneumonia-related hospitalizations to total hospitalizations, from 199.6 per 1000 inpatients in 2013 to 123.4 per 1000 inpatients in 2021 (*P* < 0.01, Fig. 2**-**A). In addition to the number of pneumonia-related deaths, the pneumonia-related in-hospital case fatality decreased year by year, from 50.2 per 1000 inpatients in 2013 to 23.9 per 1000 inpatients in 2022 (*P* < 0.01) (Fig. 2**-**B).

### Trends in the proportion of respiratory comorbidities or other underlying diseases

**Fig. 3:**
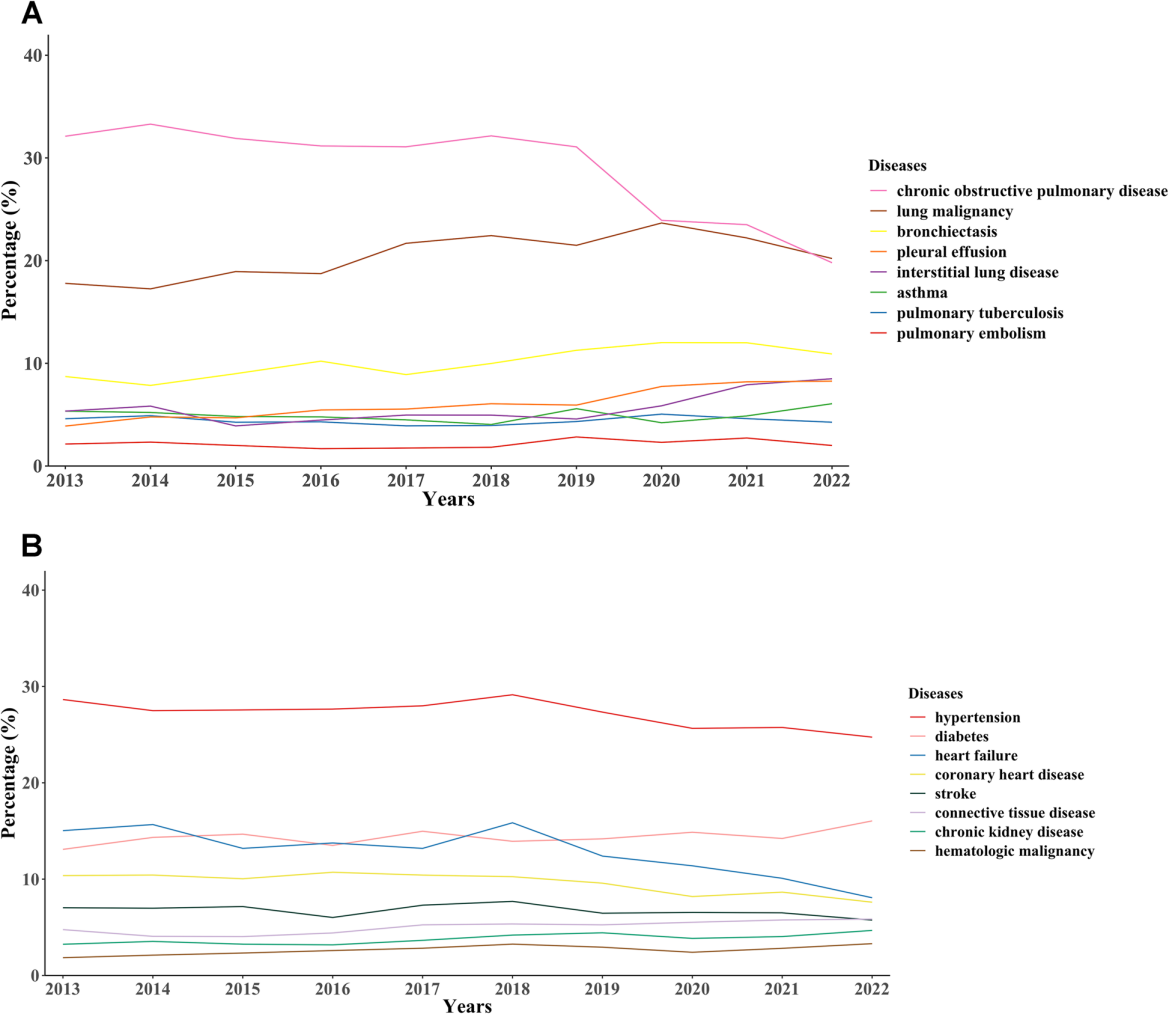
The trends in the proportion of respiratory comorbidities **A** and other underlying diseases B

Inpatients with pneumonia were often complicated by the following respiratory diseases: COPD (28%, 11,033/38870), lung malignancy (21%, 8013/38870), bronchiectasis (10%, 3990/38870), pleural effusion (6%, 2438/38870) and interstitial lung disease (6%, 2256/38870). A downward trend was observed in the proportion of pneumonia patients with COPD during the last decade (*P* < 0.01), while an increasing trend was observed in the proportion of patients with lung malignancy (*P* = 0.01) or bronchiectasis (P < 0.01) (Fig. 3**-**A). Inpatients with pneumonia were often complicated by the following underlying diseases in other systems: hypertension (27%, 10,509/38870), diabetes (14%, 5624/38870), heart failure (13%, 4868/38870), coronary heart disease (9%, 3684/38870) and stroke (7%, 2595/38870). A decreased trend was shown in the proportion of pneumonia patients with heart failure during the last decade, as well as those with hypertension or coronary heart disease (all *P* < 0.05) (Fig. 3**-**B).

### Trends in the proportion of common pathogens

**Fig. 4:**
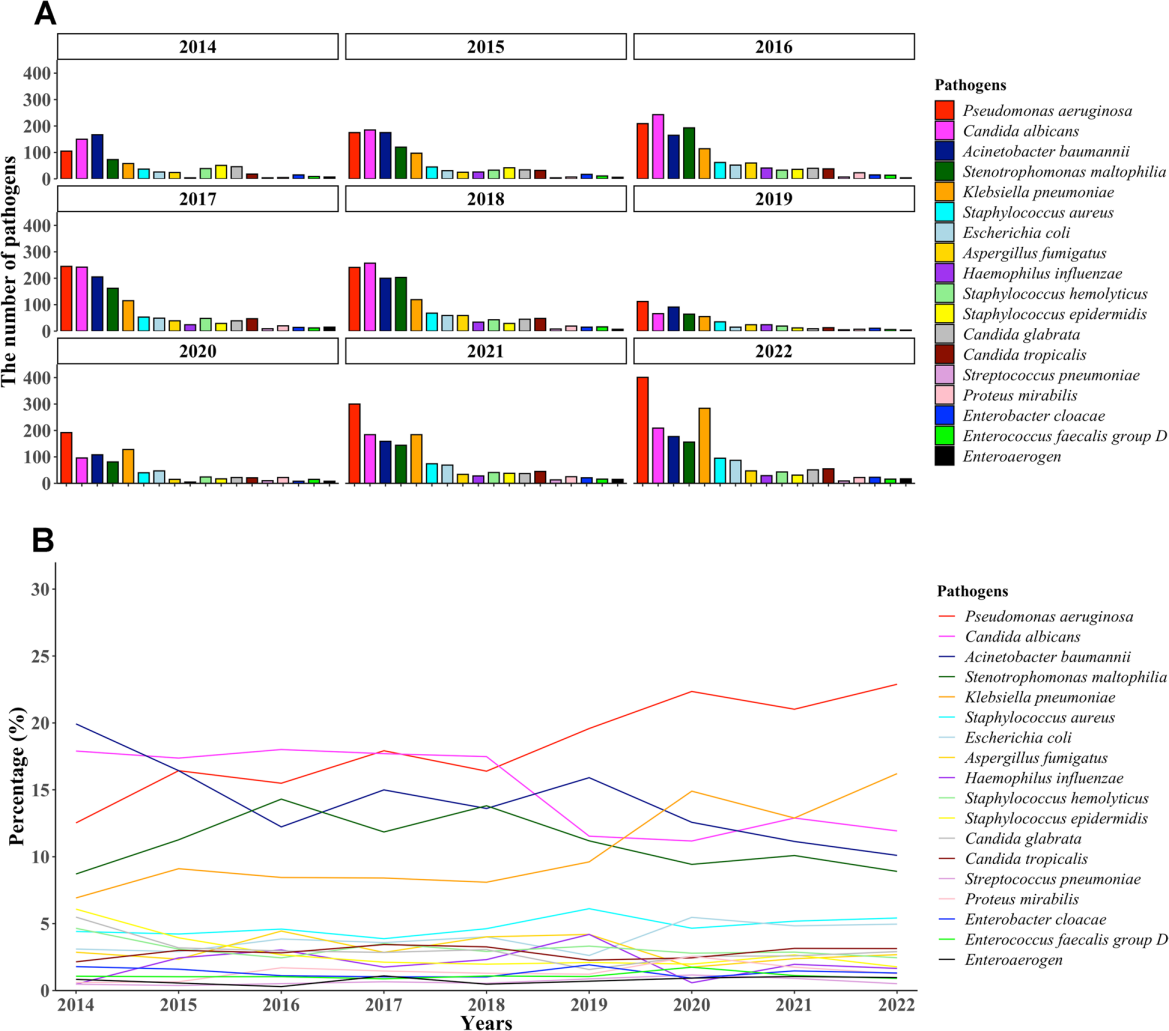
The trends in the number of isolated pathogens **A** and the percentage of isolated pathogens **B**

**Fig. 5:**
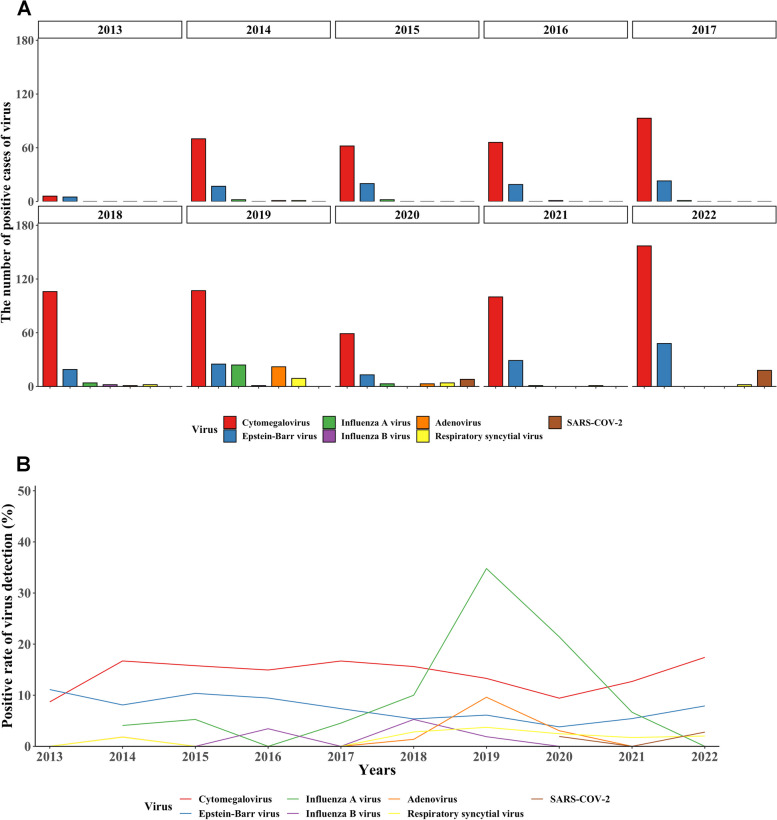
The trends in the number of positive cases of virus **A** and the positive rate of virus detection **B**

The most common isolated pathogens were *Pseudomonas aeruginosa*, *Candida albicans*, *Acinetobacter baumannii*, *Stenotrophomonas maltophilia*, *Klebsiella pneumoniae*, *Staphylococcus aureus*, *Escherichia coli*, and *Aspergillus fumigatus* (Fig. 4**-A**). The proportions of *Pseudomonas aeruginosa* and *Klebsiella pneumoniae* showed an increasing trend, while the proportions of *Candida albicans*, *Acinetobacter baumannii* and *Stenotrophomonas maltophil* showed a downward trend (all *P* < 0.05) (Fig. 4**-**B). Pairwise interaction map of pathogens can be found in Supplementary Fig. 3. Polymerase chain reaction (PCR) assay is employed routinely to detect various viruses at our center. Among the most frequently identified viruses were SARS-COV-2 (only during 2020–2022), cytomegalovirus, epstein-Barr virus, respiratory syncytial virus, adenovirus, influenza A virus, and influenza B virus (Fig. 5**-**A). Most of these viruses showed a positive detection rate of less than 20% over the past decade, with the exception of influenza A virus, which demonstrated a positive rate of 34.7% in 2019 (Fig. 5**-**B).

### Predictors for prolonged LOS in patients with pneumonia

Prolonged LOS was calculated to be 35 days in our study. After excluding the cases with missing variables, a total of 2425 cases (training set: 1682, validation set: 743) were included in the logistic regression analysis. The results of the training set showed that glucocorticoid use during hospitalization (Odd Ratio [OR] = 1.86, 95% Confidence Interval (CI): 1.14–3.06), immunosuppressant use during hospitalization (OR = 1.99, 1.14–3.46), ICU admission (OR = 16.23, 95%CI: 11.25–23.83), receiving mechanical ventilation (OR = 3.58, 95%CI: 2.60–4.97), presence of other underlying diseases (OR = 1.54, 95%CI: 1.15–2.06), and elevated procalcitonin (OR = 1.61, 95%CI: 1.19–2.19) were independent predictors for prolonged LOS. The results of the validation set were in good agreement with those of the training set (concordance index = 0.89).

## Discussion

In the context of population aging, ongoing drug-resistant pathogens and the COVID-19 epidemic, it is of interest to evaluate the changes in the epidemiological and clinical characteristics of patients with pneumonia. The main findings of the present study were the proportion of pneumonia-related hospitalizations and the in-hospital case fatality had decreased trends during the last 10 years, and that pneumonia inpatients were often complicated by chronic underlying diseases and isolated with gram-negative bacteria. Moreover, ICU admission was a significant predictor for prolonged LOS in pneumonia inpatients.

Our study showed that males accounted for the majority of inpatients with pneumonia each year, which may be attributed to the differences in physiological structure, behavior, socioeconomic status and lifestyle [13]. We found that during the last decade the middle-aged and elderly were dominant and those aged ≥58 years old accounted for more than 60% of adults with pneumonia each year, which are consistent with previous studies [14, 15]. The presence of multiple comorbidities, use of multiple drugs, immunosenescence and frailty are shown to be characteristics of the elderly and result in an increased risk of pneumonia.

In the present study, while the number of pneumonia-related hospitalizations exhibited an ascending pattern over the past decade, the proportion of patients with pneumonia has gradually reduced among all the inpatients with various diseases. This phenomenon can be partially ascribed to advances in medical technology and the convenience of medical resources, which have allowed more patients with mild or moderate pneumonia to be treated in the outpatient clinics or primary care settings. Notably, there may be a correlation between a decline in the proportion of pneumonia-related hospitalizations and a decline in the annual incidence of pneumonia, but authoritative and detailed epidemiological data are required to address this matter thoroughly. As reported previously, the incidence of pneumonia varied between countries and regions. The annual incidence of community-acquired pneumonia (CAP) in 2015 was 2.4 cases per 1000 American adults, with the highest incidence among 65–79 years (6.3/1000) and 80 years (16.4/1000) [14]. The annual incidence of CAP in Europe was estimated to be 1.07–1.2 cases/1000 persons, and 14 cases/1000 persons in those aged≥65 years [15]. However, there is a paucity of data on the pneumonia incidence in China. We speculate that a lot of factors may contribute to the regional differences in pneumonia incidence, such as socioeconomic levels, health care systems, underlying health status of individuals, and whether receiving streptococcus pneumonia vaccine and influenza vaccine. On the other hand, we observed that both the pneumonia-related deaths and the in-hospital case fatality had a downward trend, which may be inseparable from advances in medical technology, including emerging molecular diagnostic methods, improved antimicrobial drugs, powerful life-support instruments (e.g. extracorporeal membrane oxygenation) and optimized treatment strategies. However, as the population ages, the mortality of the elderly caused by pneumonia should not be ignored. As shown in the latest GBD study, LRTIs caused 2.49 million deaths in 2019 globally, with 1.23 million deaths in the population older than 70 years old [2]. Similarly, another study reported 1.1 million pneumonia-related hospital deaths in the elderly in 2015 [16]. Moreover, the mortality rate was shown to be associated closely with increasing age, with 5% for CAP patients aged ≥65 years, 8% for patients aged 65–79 years, and 14% for patients ≥80 years [17].

Our results suggested that patients with pneumonia were complicated by various chronic underlying diseases, similar to a previous study in which respiratory diseases, diabetes mellitus, cardiovascular disease, and chronic liver disease were the most common comorbidities that may increase the risk of CAP [15]. COPD, a chronic respiratory disease with high morbidity and mortality, should be highlighted here. Globally, 212.3 million cases of COPD were reported in 2019, with COPD accounting for 3.3 million deaths [18]. Previous studies showed there was an approximately 18-fold greater incidence of CAP in COPD patients than in those without COPD [19] and that COPD was an important cause of a 30-day readmission in patients with pneumonia [20]. In addition to COPD, the relationship between pneumonia and cardiovascular diseases also deserves attention. On the one hand, preexisting heart failure was identified as a risk factor for pneumonia, and pulmonary congestion promoted the growth of common respiratory pathogens [21]. On the other hand, pneumonia may cause or aggravate heart failure, myocarditis, arrhythmia, ischemia, and infarction [21], and inpatients with pneumonia had increased short-and long-term risks of cardiovascular diseases [22]. Interestingly, we found that the percentages of COPD, heart failure, hypertension and coronary heart disease showed downward trends over the past decade, particularly in the past 3 years. This may be due to the effective management of these chronic diseases, and may also be related to the changes in people’s behavior during the COVID-19 pandemic, such as wearing masks, going out less, consulting a physician via internet medical service and obtaining medicines for daily maintenance online. We also observed that the percentage of lung malignancy had an upward trend, which could be explained by the fact that more and more patients are being screened or examined for lung cancers.

Although initial antimicrobial therapy of pneumonia is often performed empirically, the identification of causative pathogens is of great importance, especially in severe cases. The present study revealed the predominant isolated pathogens were *Pseudomonas aeruginosa*, *Candida albicans*, *Acinetobacter baumannii*, *Neurotrophomonas maltophilia*, *Klebsiella pneumoniae*, and *Staphylococcus aureus*, which have been reported to be more common in hospital-acquired pneumonia (HAP) than in CAP [1]. By contrast, *Streptococcus pneumoniae* and *Haemophilus influenzae,* which are very common in CAP, accounted for a small proportion of the isolated pathogens. The above results may be interpreted as followings. Initially, among the inpatients with pneumonia in this study, a substantial proportion of them have received multiple antibiotics or even mechanical ventilation in other hospitals, which may affect the detection sensitivity and species of pathogens. For instance, these individuals exhibited a higher likelihood of being infected with gram-negative bacteria. Furthermore, the isolated pathogens may be contaminating bacteria or colonizing bacteria, such as *Candida albicans* and *Acinetobacter baumannii*, so we should take into account the other clinical variables when determining whether an isolated pathogen is causative. Additionally, pneumococcal polysaccharide vaccines may provide protection against invasive pneumococcal disease [23], and reduce the risk of *Streptococcus pneumoniae* infection. Although the patients with viral pneumonia were less common (*n* = 458) compared with bacterial pneumonia, we should keep an eye on the detection of the most common viruses, including SARS-COV-2, cytomegalovirus, epstein-Barr virus, respiratory syncytial virus, adenovirus, influenza A virus, and influenza B virus.

Our study revealed that glucocorticoid use during hospitalization, immunosuppressant use during hospitalization, ICU admission, receiving mechanical ventilation, presence of chronic underlying diseases, and elevated procalcitonin may contribute to a prolonged LOS in pneumonia inpatients. Among the above factors, ICU admission was the most prominent factor, which often indicates a very severe illness. As reported previously, the causes of prolonged LOS were classified as pneumonia-related, complications, instability of the underlying disease, and non-clinical factors [24]. Pneumonia severity index high risk grade, positive blood culture, ICU admission, multiple lobe involvement, and alcohol consumption were shown to be independently associated with prolonged LOS [25]. Hypoxemia and pleural effusion were identified as predictors for LOS in all patients with pneumonia, while diastolic blood pressure, multiple lobe involvement, and hypoalbuminemia were predictors for LOS in high-risk patients with pneumonia [26]. However, the effect of glucocorticoid use during hospitalization on LOS and other outcomes in patients with pneumonia remains controversial. A study by Melanie Lloyd et al. showed that adjuvant prednisolone acetate (50 mg/day for 7 days) led to an increased risk of gastrointestinal bleeding without reducing LOS and mortality [27]. In another study, dexamethasone (5 mg/d for 4 days) in addition to antibiotics could shorten the LOS in non-immunocompromised patients with CAP [28]. We speculate that the differences may be related to the type, dose and administration route of glucocorticoid, as well as other therapies and selected participants. In summary, the prolonged LOS may be multifactorial and complex, and it is imperative to prioritize patients with more severe conditions and adopt the controversial treatment circumspectly.

A particular strength of this study was using a NLP system to automatically extract a large amount of clinical information from EHR, which may help promote the application of AI in the real-world data in the future. Another strength was that the data from our center were somewhat nationally representative, because our hospital has been the largest respiratory medical centre in China since 2009 and has treated more than 240,000 inpatients in the past 10 years. However, our study also has some limitations. First, the conclusions drawn from this study may not be entirely applicable to other countries or regions due to the differences in socioeconomic levels, health care systems, and health status of patient population, especially during the year 2020–2021 when the pattern and management of COVID-19 in China was very different from the rest of the world. For example, during this period, patients with COVID-19 were mainly admitted to designated hospitals for infectious diseases, and strict epidemic prevention and control against COVID-19 have limited the flow of patients with other diseases to some extent. This may partly explain why the number of pneumonia-related hospitalizations decreased in 2020 and 2021. Second, since a considerable proportion of inpatients have complicated medical history before adimitted to our hospital, we did not accurately distinguish between CAP and HAP. Third, the detection efficiency of pathogens has improved recently due to diagnostic techniques such as meta-gene second-generation sequencing (mNGS) [29, 30]. However, since mNGS are often tested by third-party institutions, our NLP system could not extract such data outside the hospital, so the pathogens presented in this study were incomplete somewhat.

## Conclusion

Despite an increasing trend in the number of pneumonia-related hospitalizations, both the proportion of pneumonia-related hospitalizations in total hospitalizations and the in-hospital case fatality showed decreased trends during the last decade. Inpatients with pneumonia were more common in the elderly. They were often accompanied by COPD, cardiovascular diseases, lung malignancy, bronchiectasis and diabetes, and isolated with gram-negative bacteria. ICU admission was a significant predictor for prolonged LOS in pneumonia patients. However, more relevant studies from other countries or regions are needed to provide comprehensive understanding of the clinical and epidemiological changes in patients with pneumonia.

### Supplementary Information

**Additional file 1: Supplementary Figure 1. **The trends in gender in hospitalize patients with pneumonia from 2013 to 2022.

**Additional file 2: Supplementary Figure 2. **The trends in age composition in hospitalized patient with pneumonia from 2013 to 2022.

**Additional file 3: Supplementary Figure 3. **Pair wise interaction map of the isolated pathogens.

## Data Availability

If required reasonably, all datasets generated and analyzed during the study can be achieved from corresponding author Jinping Zheng.

## Abbreviations

AI: Artificial intelligence
CAP: Community-acquired pneumonia
COPD: Chronic obstructive pulmonary disease
COVID-19: Coronavirus disease 2019
EHR: Electronic health record
GBD: Global Burden of Disease
HAP: Hospital-acquired pneumonia
LOS: Length of stay
LRTIs: Lower respiratory tract infections
mNGS: Meta-gene second-generation sequencing
NLP: Natural language processing
PCR: Polymerase chain reaction
