# Supplementary figures and images for: Hospitalization, case fatality, comorbidities, and isolated pathogens of adult inpatients with pneumonia from 2013 to 2022: a real-world study in Guangzhou, China
Source: BMC Infect Dis. 2024 Jan 2;24:2. doi: 10.1186/s12879-023-08929-y (PMC10759351; doi:10.1186/s12879-023-08929-y)

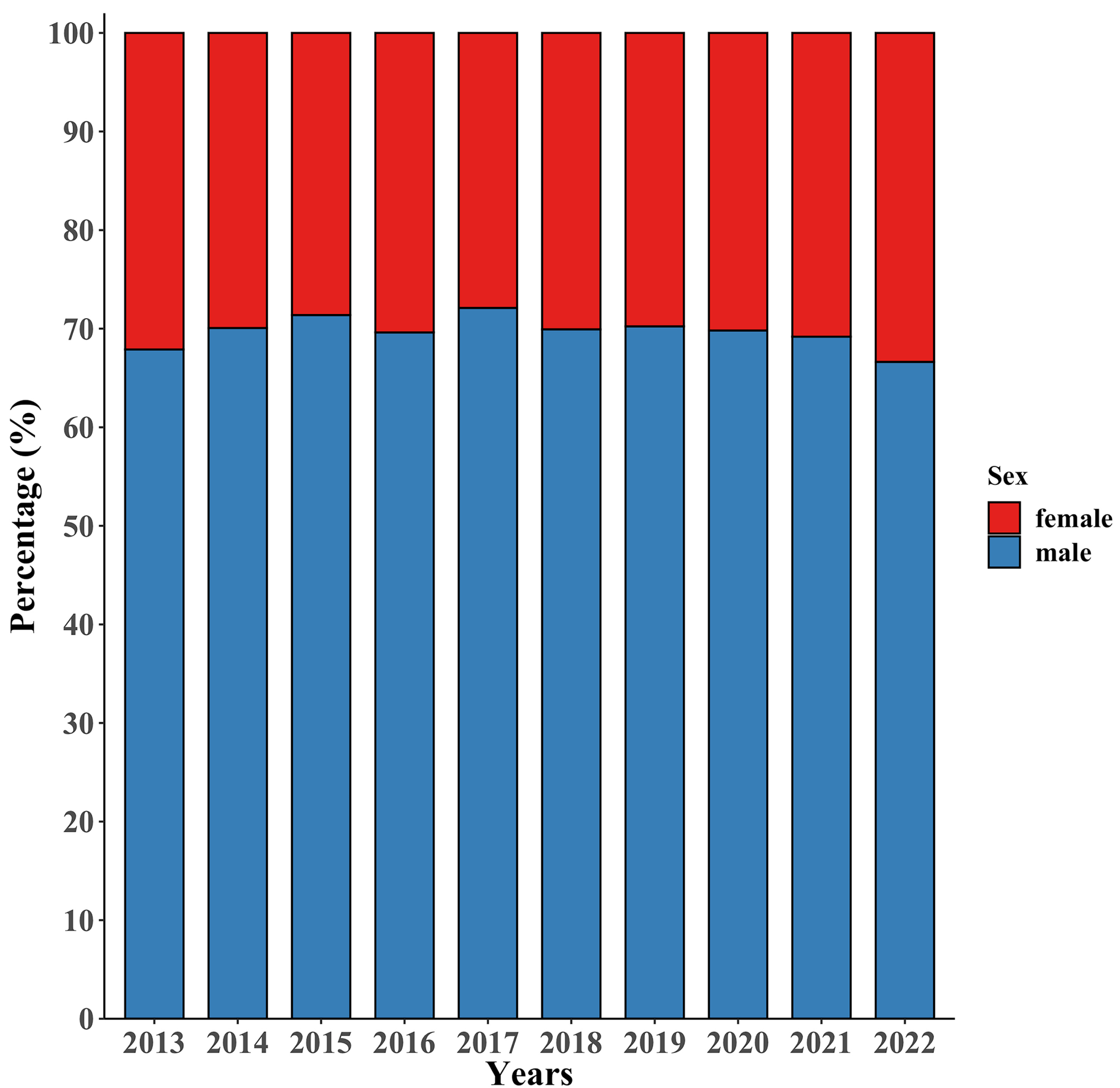

**Supplementary Figure1** The trends in gender in hospitalized patients with pneumonia from 2013 to 2022.

Supplement: Supplementary file 1 — Additional file 1: Supplementary Figure 1. The trends in gender in hospitalize patients with pneumonia from 2013 to 2022. [file 12879_2023_8929_MOESM1_ESM.pdf]

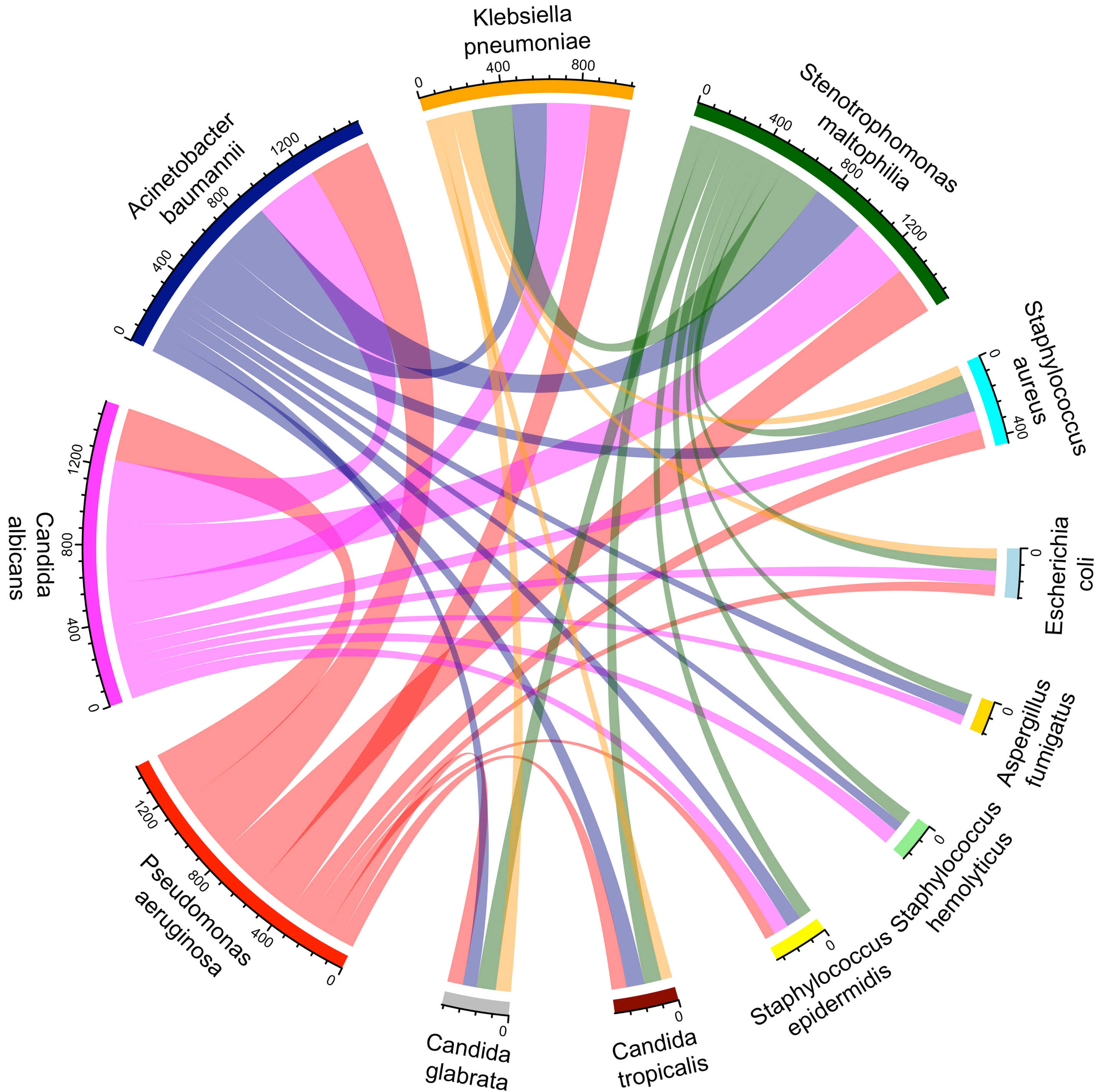

**Supplementary Figure 3** Pair wise interaction map of the isolated pathogens

Supplement: Supplementary file 3 — Additional file 3: Supplementary Figure 3. Pair wise interaction map of the isolated pathogens. [file 12879_2023_8929_MOESM3_ESM.pdf]
