# Supplementary material for: Hospitalization, case fatality, comorbidities, and isolated pathogens of adult inpatients with pneumonia from 2013 to 2022: a real-world study in Guangzhou, China
Source: BMC Infect Dis. 2024 Jan 2;24:2. doi: 10.1186/s12879-023-08929-y (PMC10759351; doi:10.1186/s12879-023-08929-y)
Supplement: Supplementary file 2 — Additional file 2: Supplementary Figure 2. The trends in age composition in hospitalized patient with pneumonia from 2013 to 2022. [file 12879_2023_8929_MOESM2_ESM.pdf]

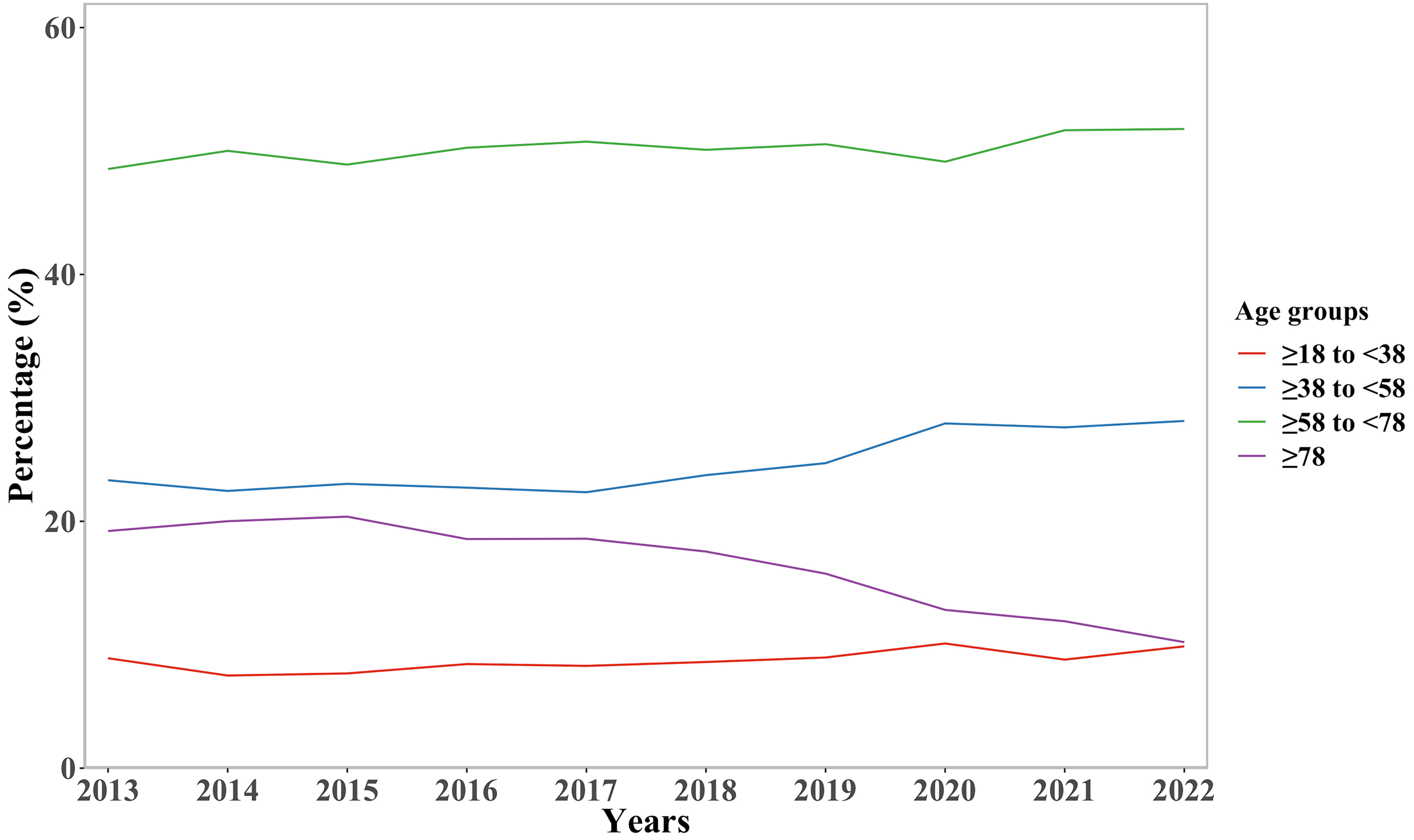

Supplementary Figure 2 The trends in age composition in hospitalized patients with pneumonia from 2013 to 2022.
